# Pharmacological Strategies for Preventing Postoperative Recurrence in Crohn’s Disease: A Systematic Review and Network Meta-Analysis of Randomized Controlled Trials

**DOI:** 10.3390/medicina62050883

**Published:** 2026-05-05

**Authors:** Wei Chen, Xin Tong, Yuhang Liu, Xi Zhang, Siying Zhu, Yanhua Zhou, Yongdong Wu, Ye Zong

**Affiliations:** 1 Beijing Key Laboratory of Early Gastrointestinal Cancer Medicine and Medical Devices, Department of Gastroenterology, Beijing Friendship Hospital, Capital Medical University, 95 Yong-An Road, Xi-Cheng District, Beijing 100050, China; wchenYY2020@163.com (W.C.); tong172607@163.com (X.T.); lyhccmu506@163.com (Y.L.); zhangxi1114@ccmu.edu.cn (X.Z.); zhouyanhuazyh@sina.com (Y.Z.); wuyongdong2018@sina.com (Y.W.); 2 National Clinical Research Center for Digestive Disease, State Key Laboratory of Digestive Health, Beijing Friendship Hospital, Capital Medical University, 95 Yong-An Road, Xi-Cheng District, Beijing 100050, China

**Keywords:** Crohn’s disease, postoperative recurrence, pharmacological prevention, network meta-analysis, therapeutic efficacy

## Abstract

*Background and Objectives*: Despite surgical intervention for remission, recurrence is nearly inevitable in patients with Crohn’s disease (CD). While several maintenance therapies are available, the optimal strategy for preventing postoperative recurrence remains uncertain. *Materials and Methods*: This systematic review and network meta-analysis included placebo-controlled or head-to-head randomized controlled trials (RCTs) from MEDLINE, Embase, and Cochrane Central up to 4 July 2024. Studies assessed maintenance therapies for CD after curative resection. Data were extracted from intention-to-treat (ITT) and per-protocol (PP) analyses separately. The primary outcomes were endoscopic and clinical relapse. A Bayesian network meta-analysis provided risk ratios (RRs) and 95% confidence intervals (CIs). This study is registered with PROSPERO (CRD42024629013). *Results*: From 1492 screened records, 45 randomized controlled trials met the inclusion criteria. Compared with placebo, clinically significant prevention of clinical recurrence was achieved with adalimumab (RR = 0.17; GRADE High), nitroimidazoles (RR = 0.35; High), infliximab (RR = 0.59; Moderate), thiopurine analogs (RR = 0.41; Moderate), and high-dose mesalamine (RR = 0.74; High), while azathioprine-metronidazole combination therapy demonstrated superior efficacy to azathioprine monotherapy. For endoscopic recurrence mitigation, therapeutic efficacy was confirmed for adalimumab (RR = 0.24; Low), infliximab (RR = 0.32; Moderate), vedolizumab (RR = 0.36; Low), and thiopurine analogs (RR = 0.64; Moderate). *Conclusions*: This network meta-analysis establishes pharmacological hierarchies for preventing postoperative Crohn’s disease recurrence. Adalimumab is the most effective monotherapy for clinical recurrence prevention, while combination therapies of adalimumab/azathioprine plus nitroimidazole show superior efficacy. For endoscopic recurrence prevention, adalimumab also ranks as the most effective intervention. These findings guide therapy selection but require validation for newer agents through randomized trials.

## 1. Introduction

Crohn’s disease (CD), a chronic inflammatory bowel disorder characterized by transmural inflammation of any gastrointestinal segment, predominantly affects the terminal ileum and colon. Its relapsing-remitting course manifests with debilitating symptoms including abdominal pain, diarrhea, and weight loss, substantially impairing quality of life [1].

Surgical intervention remains inevitable for 39–82% of CD patients to address complications such as strictures or penetrating lesions [2]. Regrettably, this kind of procedure does not offer a definitive remedy for CD. A significant number of patients will experience postoperative recurrence (POR) [3], which can exert a substantial impact on both their personal well-being and socioeconomic circumstances. Endoscopic recurrence typically precedes clinical symptoms, underscoring the need for proactive pharmacological prophylaxis [4].

Current strategies to mitigate POR risk remain contentious. Anti-TNF agents (infliximab, adalimumab) demonstrate efficacy in reducing endoscopic recurrence rates [5,6,7]. Comparative effectiveness against newer biologics (ustekinumab, vedolizumab) remains unclear due to limited randomized controlled trials [4]. While ECCO guidelines conditionally recommend anti-TNF therapy for high-risk patients [4], ACG emphasizes individualized decision-making given heterogeneous safety profiles and cost-effectiveness considerations [8]. Immunomodulators (thiopurines) show moderate benefit but are limited by toxicity, and evidence for small-molecule agents (JAK inhibitors) remains nascent [3,4].

It has been several years since the last comprehensive Cochrane network meta-analysis in 2019 [3]. From 2019 to 2024, a wealth of new evidence regarding drug comparisons for preventing postoperative recurrence (POR) has emerged [9,10]. This network meta-analysis (NMA) systematically updates and evaluates randomized controlled trials (RCTs) to address two critical gaps: (1) the comparative efficacy of different therapies in preventing POR; (2) the safety trade-offs between drug classes. By synthesizing direct and indirect evidence through Bayesian frameworks, our findings aim to refine evidence-based POR prophylaxis protocols.

## 2. Materials and Methods

Our analysis methods and inclusion criteria were developed in accordance with the recommendations outlined in the Preferred Reporting Items for Systematic Reviews and Meta-Analyses (PRISMA) guidelines [11,12]. Additionally, the study protocol was registered in the PROSPERO database (registration number: CRD42024629013).

**Study Inclusion and Exclusion**

Studies meeting the criteria outlined in the sections “Design of Eligible Studies,” “Eligible Participants,” and “Types of Interventions,” and reporting at least one of the outcomes specified under “Primary and Secondary Outcomes,” were included. No restrictions were applied regarding sample size, follow-up duration, or reporting format (full-text or abstract). The detailed inclusion and exclusion criteria are provided below.

**Design of Eligible Studies**

Only randomized controlled trials (RCTs) were included. Cross-sectional studies, meta-analyses, reviews, case reports, editorials, preclinical studies, or other irrelevant studies were excluded. Additionally, studies involving duplicate cohorts were excluded.

**Eligible Participants**

Participants included in the analysis were required to have Crohn’s disease (CD), diagnosed based on established clinical, radiological, or endoscopic criteria. Only patients in remission following surgery, confirmed by pathological evidence of no residual lesions, were eligible. Surgical resection must have been performed within six months prior to the initiation of maintenance therapy.

**Types of Interventions**

No restrictions were placed on the type of pharmaceutical interventions used for the prevention of postoperative recurrence in Crohn’s disease. Trials evaluating any pharmaceutical therapy, including oral or topical corticosteroids, 5-ASA agents, purine analogs, TNF-α antagonists, other biologic agents, probiotics, antibiotics, or other interventions, were considered eligible. Studies were included if participants received treatment for at least three months.

**Primary and Secondary Outcomes**

The primary outcomes were rigorously defined as clinical recurrence and endoscopic recurrence. Treatment discontinuation attributable to documented adverse pharmacological reactions constituted secondary outcome measures. To ensure data integrity, studies demonstrating ambiguous operational definitions of either recurrence patterns or toxicity profiles underwent systematic exclusion. Analytical outcomes principally incorporated intention-to-treat population estimates, with per-protocol cohort findings subsequently presented through sensitivity analyses.

**Search Methods for Identification of Studies**

Relevant studies were identified through systematic searches of Medline via Ovid, Embase via Embase.com, the Cochrane Central Register of Controlled Trials (CENTRAL), and the ClinicalTrials.gov registry from their inception to July 2024. The detailed search strategy, including free-text terms and MeSH terms used in Medline, is provided in Supplementary Table S1. Additionally, bibliographies of relevant reviews and systematic reviews were manually screened.

**Assessment of Risk of Bias in Included Studies**

Two independent reviewers (CW and TX) assessed the risk of bias for all eligible studies using the Revised Cochrane Risk of Bias (RoB 2) tool for randomized controlled trials. Discrepancies in quality assessment were resolved through consensus discussions with the principal investigator (ZY).

**Data Collection and Analysis**

Study eligibility was independently evaluated by two reviewers (CW and TX) through an initial screening of titles and abstracts. Full-text articles of studies deemed potentially eligible were then reviewed to confirm final inclusion. Any disagreements were resolved in consultation with the senior author (ZY).

**Data extraction and management**

Data were extracted into an Excel extraction form by one investigator and double-checked by another:

(1) Characteristics of the study: Title, first author, publication year, country, study design, reporting forms, and sample size.
(2) Population characteristics: Gender, age, surgical history, smoking status, disease duration, disease distribution, disease behaviors (perforation, anorectal involvement, and extensive bowel involvement), anastomosis site, anastomosis method, and pathological features (positive margin proportion, margin plexitis proportion, and granuloma).
(3) Intervention characteristics: All information regarding study medications and concomitant medications was thoroughly documented.
(4) Follow-up: Length of follow-up.
(5) Outcomes: Definitions of endoscopic relapse (ER) and clinical relapse (CR), number of ER and CR, number of participants who discontinued treatment due to adverse drug reactions (ADRs).

**Dealing with missing data**

For incomplete dichotomous outcome parameters, intention-to-treat analytic protocols were implemented with the a priori assumption that all discontinued participants represented therapeutic failure cases. This conservative imputation strategy was deemed methodologically sound for assessing both clinical recurrence and endoscopic recurrence.

**Measures of Treatment Effect**

All analyses were performed using R software (version 4.4.2; R Foundation for Statistical Computing, 2024) with the “gemtc” package for Bayesian network meta-analysis [13,14]. A random-effects model was implemented to account for heterogeneity across studies. Treatment effects were summarized as risk ratios (RRs) with 95% confidence intervals (CIs), where an RR < 1.0 indicated a lower recurrence risk in the experimental group relative to the comparator.

**Treatment Hierarchy Quantification**

Intervention efficacy rankings were determined through Bayesian posterior probability analysis. For each treatment, the Surface Under the Cumulative Ranking curve (SUCRA) was calculated by integrating cumulative ranking distributions, with values ranging 0–100% (100% representing maximal therapeutic superiority). This metric synthesizes multidimensional ranking probabilities (best-to-worst) into scalar efficacy estimators, enabling evidence-based prioritization of clinical strategies.

**Assessment of Consistency**

Node-splitting analysis was performed using “gemtc” with binomial likelihood and log-link function. Closed-loop comparisons were systematically identified through mtc.nodesplit(). Four Markov chains completed 5000 adaptation iterations followed by 20,000 sampling cycles. Direct–indirect evidence discrepancies were quantified using Bayesian *p*-values, with statistical significance threshold at α = 0.05. Analytical implementation strictly followed the mtc.nodesplit() framework.

**Assessment of Heterogeneity**

Heterogeneity in the network was quantified using Bayesian variance component analysis through the “mtc.anohe” function in the “gemtc” package. This approach decomposes the total between-study variance (τ^2^) into its posterior distribution, with 95% credible intervals (CIs) reflecting uncertainty. The magnitude of heterogeneity was further characterized by the Bayesian analog of I^2^, calculated as τ^2^/(τ^2^ + σ^2^) × 100%, where σ^2^ represents within-study variance. A posterior probability > 95% for I^2^ exceeding 50% was considered indicative of substantial heterogeneity.

To identify heterogeneity sources, we implemented hierarchical model extensions incorporating covariates through Markov chain Monte Carlo (MCMC) sampling. The relative contribution of each covariate to τ^2^ was estimated via variance partition coefficients (VPCs), with a threshold of >20% VPC deemed clinically meaningful.

**Meta Regression**

Univariate meta-regression was conducted to investigate potential sources of heterogeneity, incorporating covariates including sample size, follow-up duration, male proportion, age, surgical history, smoking prevalence, disease duration, and proportions of severe disease manifestations (intestinal perforation, anorectal involvement, and extensive intestinal involvement). This analytical approach systematically evaluated the moderating effects of methodological and clinical variables on outcome variations across studies.

**Sensitivity** **Analysis**

Two methodological safeguards were systematically implemented: (1) When the heterogeneity test showed I^2^ < 25%, fixed-effect models were performed to verify result consistency. (2) To further validate the stability of the results, a sensitivity analysis was conducted using the frequentist approach implemented in CINeMA (Confidence In Network Meta—Analysis), which is based on the “netmeta” package in R. (3) Per-protocol cohort validation was performed through complete-case analysis excluding protocol violators. All analytical frameworks maintained identical binomial likelihood specifications and logit-link functions to ensure cross-model comparability.

**Assessment of Reporting Bias**

Possible selective publication tendencies were evaluated through graphical symmetry analysis of effect size dispersion relative to precision metrics (funnel plot methodology). Concurrently, the systematic assessment of financial conflict influences (industry-sponsored vs. independent research) and underpowered study artifacts was conducted through stratified sensitivity modeling. Asymmetric distribution patterns across precision quantiles were quantified using Egger’s regression coefficient with permutation testing (1000 iterations).

**Assessment of the Certainty of the Evidence**

The methodological rigor of network meta-analysis outcomes was appraised using the Confidence in Network Meta-Analysis (CINeMA) framework, a publicly accessible open access platform (“https://cinema.ispm.unibe.ch/ (accessed on 3 March 2025)”) grounded in Grading of Recommendations Assessment, Development and Evaluation (GRADE) principles [15,16]. This structured approach facilitates systematic assessment across six critical dimensions: (a) Within-study bias, (b) Reporting bias, (c) Indirectness, (d) Imprecision, (e) Heterogeneity, and (f) Incoherence. Each criterion underwent three-tier classification: “no concern”, “some concern”, or “major concern”, following standardized GRADE derivation protocols. The reviewer (CW) then chooses to summarize judgments across domains using the 4 levels of confidence of the GRADE approach: very low, low, moderate, or high.

## 3. Results

**Figure 1:**
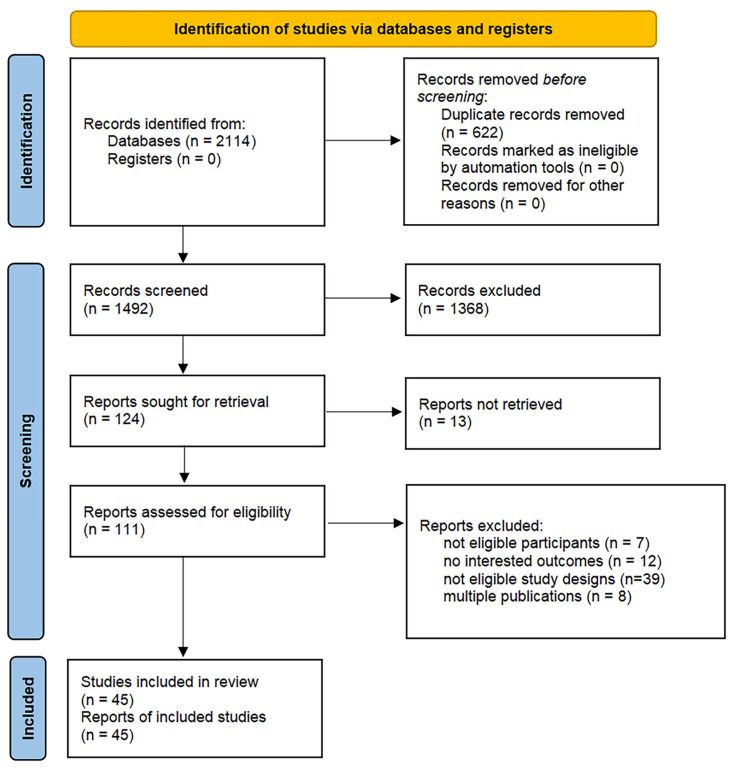
PRISMA flow diagram of the literature search and study selection process.

The systematic search identified 2114 candidate records, with 45 randomized controlled trials (RCTs) [5,6,7,9,10,17,18,19,20,21,22,23,24,25,26,27,28,29,30,31,32,33,34,35,36,37,38,39,40,41,42,43,44,45,46,47,48,49,50,51,52,53,54,55] ultimately meeting predefined eligibility criteria following rigorous screening (Figure 1). Critical appraisal using the Cochrane ROB2 tool demonstrated significant methodological variability among included trials (Supplementary File S1), with final ratings distributed as low risk (*n* = 23), some concerns (*n* = 17), and high risk (*n* = 5) of bias.

The evidence base spanned a 48-year investigation period (1976–2024), with early-phase research focusing on 5-aminosalicylates, nitroimidazoles, thiopurine analogs, and probiotics. Biologic therapies, including infliximab (IFX), adalimumab (ADA), and vedolizumab (VDZ), entered clinical investigation post-2009, though notable evidence gaps persisted for ustekinumab (UST) and Janus kinase (JAK) inhibitors. Ancillary pharmacological interventions encompassed corticosteroids, cyclosporine, Tripterygium glycosides (TG), vitamin D, and interleukin-10 (IL-10), with limited exploration of combination regimens and administration optimization strategies.

The intention-to-treat (ITT) cohorts exhibited marked heterogeneity in sample sizes, with a median enrollment of 40 participants (interquartile range [IQR] 22–62.5; minimum-maximum: 7–170). Enrolled populations comprised predominantly young adults (mean age: 35.0 ± 2.8 years) with balanced sex distribution (male proportion: median 51.0% [IQR 43.0–59.5%]). Surveillance intervals were standardized at a median of 12 months [IQR 12–18], with 64.4% (29/45) of studies reporting both clinical and endoscopic recurrence data, compared to exclusive clinical (11.1%, 5/45) or endoscopic (20.0%, 9/45) relapse documentation. Detailed trial-level characteristics are systematically tabulated in Table 1.

### 3.1. Comparative Efficacy for Clinical Recurrence Prevention

**Figure 2:**
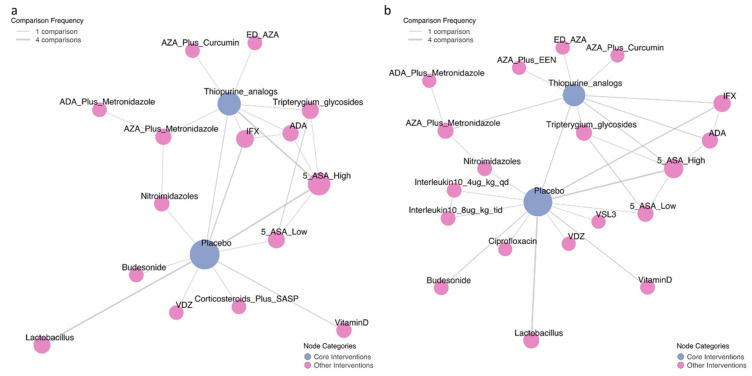
Network geometry of randomized controlled trials evaluating pharmacological interventions for preventing postoperative recurrence in Crohn’s disease. The size of nodes corresponds to the number of patients receiving each treatment, and the thickness of edges represents the number of direct comparisons between treatments. (**a**) Network for clinical recurrence prevention (34 trials, 3689 patients). (**b**) Network for endoscopic recurrence prevention (39 trials, 3748 patients). Abbreviations: AZA, Azathioprine; ED-AZA, Endoscopy-driven AZA; ADA, Adalimumab; 5 ASA, 5-Aminosalicylic Acid; IFX, Infliximab; EEN, Early Enteral Nutrition; VDZ, Vedolizumab. VSL#3—Each sachet contains 900 billion viable bacteria, comprising 4 strains of Lactobacillus, 3 strains of Bifidobacterium, and 1 strain of Streptococcus salivarius subspecies thermophilus.

**Figure 3:**
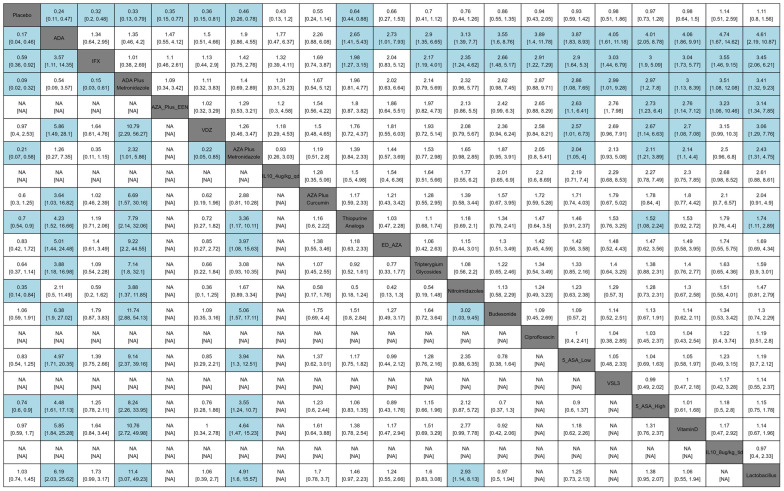
League table of comparative efficacy for postoperative recurrence prevention in Crohn’s disease. Drug names are positioned along the diagonal cells. Numerical values (risk ratios with 95% confidence intervals) in each cell indicate the comparative efficacy between the right-listed intervention and the upper-listed counterpart. The upper triangular matrix displays endoscopic recurrence outcomes, whereas the lower triangular matrix presents clinical recurrence outcomes. Blue-shaded cells denote statistically significant outcomes (*p* < 0.05). Abbreviations: ADA, Adalimumab; IFX, Infliximab; AZA, Azathioprine; EEN, Early Enteral Nutrition; VDZ, Vedolizumab; AZA, Azathioprine; IL-10, Interleukin-10; ED-AZA, Endoscopy Driven AZA; 5 ASA, 5-Aminosalicylic Acid. VSL#3—Each sachet contains 900 billion viable bacteria, comprising 4 strains of Lactobacillus, 3 strains of Bifidobacterium, and 1 strain of Streptococcus salivarius subspecies thermophilus.

**Figure 4:**
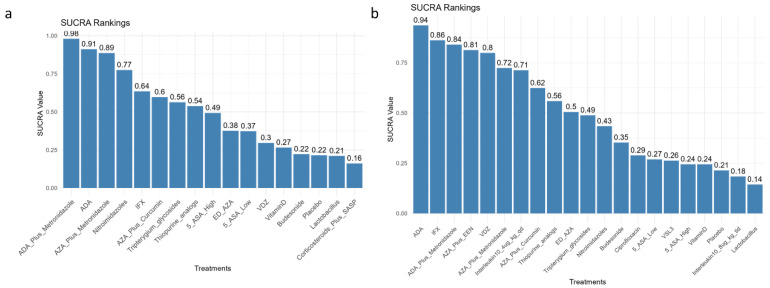
Surface under the cumulative ranking curve (SUCRA) values for pharmacological interventions in preventing postoperative recurrence of Crohn’s disease. (**a**) SUCRA rankings for clinical recurrence prevention. (**b**) SUCRA rankings for endoscopic recurrence prevention. Higher SUCRA values (0–100%) indicate a greater probability of treatment being among the most effective options. Abbreviations: ADA, Adalimumab; AZA, Azathioprine; IFX, Infliximab; 5 ASA, 5-Aminosalicylic Acid; ED-AZA, Endoscopy-driven AZA; VDZ, Vedolizumab; IFX, Infliximab; EEN, Early Enteral Nutrition. VSL#3—Each sachet contains 900 billion viable bacteria, comprising 4 strains of Lactobacillus, 3 strains of Bifidobacterium, and 1 strain of Streptococcus salivarius subspecies thermophilus.

The network meta-analysis incorporated 34 randomized trials involving 3689 post-operative Crohn’s disease patients evaluating clinical recurrence prevention. Therapeutic comparisons predominantly utilized placebo or thiopurine analogs as reference arms (Figure 2). Bayesian hierarchical modeling under random-effects assumptions (Figure 3, lower-left matrix) demonstrated superior efficacy of adalimumab plus metronidazole versus placebo (RR = 0.09, 95%CI 0.02–0.32; SUCRA = 0.98; GRADE Moderate), followed sequentially by: adalimumab monotherapy (RR = 0.17, 0.04–0.46; SUCRA = 0.91; GRADE High), azathioprine/metronidazole combination (RR = 0.19, 0.07–0.58; SUCRA = 0.89; GRADE Moderate), nitroimidazoles (RR = 0.35, 0.14–0.84; SUCRA = 0.77; GRADE High), infliximab (RR = 0.59, 0.36–0.92; SUCRA = 0.64, GRADE Moderate), thiopurine analogs (RR = 0.70, 0.54–0.90; SUCRA = 0.54; GRADE Moderate), and high-dose mesalamine ≥ 3 g/d (RR = 0.74, 0.60–0.90; SUCRA = 0.49; GRADE High). It was found that among the effective drugs mentioned above, ADA was superior to IFX with an RR of 3.57 (IFX vs. ADA, 95%CI: 1.11–14.35; GRADE Low) and thiopurine analogs with an RR of 4.23 (thiopurine vs. ADA, 95%CI: 1.52–16.66; GRADE Moderate). Additionally, the combination of ADA and metronidazole was more effective than metronidazole monotherapy, presenting an RR of 3.88 (95%CI: 1.37–11.85, GRADE Moderate). The AZA-metronidazole combination therapy demonstrated superior efficacy compared to AZA monotherapy, evidenced by a risk ratio of 3.36 (95% CI: 1.17–10.11; GRADE Moderate). Vedolizumab showed non-significant efficacy (RR = 0.97, 0.40–2.53; SUCRA = 0.30; GRADE Low), with complete SUCRA distributions detailed in Figure 4a.

Model consistency was validated through node-splitting analysis (all direct–indirect comparison *p* > 0.05, Supplementary Figure S1a), indicating negligible local inconsistency. Global heterogeneity remained minimal (I^2^ = 4.47%), with concordant effect estimates between fixed-effect (Supplementary Figure S2) and random-effects models.

As shown in Supplementary File S2, the outcomes of the sensitivity analysis grounded in the frequentist approach are in accordance with those obtained by the Bayesian method above.

Per-protocol evaluation (18 trials, *n* = 1225) revealed substantial concordance between pharmacological efficacy risk ratios (RRs) and preceding intention-to-treat findings. Notably, azathioprine combined with metronidazole achieved statistical significance versus placebo controls (*p* < 0.05), whereas all other therapeutic interventions failed to demonstrate clinically meaningful differentiation (Supplementary Figure S3).

Begg’s funnel plot analysis and Egger’s regression test (*p* = 0.74) did not reveal statistically significant asymmetry, suggesting no substantial evidence of publication bias across the included studies.

The evidence levels of all drug comparison results are summarized in Supplementary File S3.

### 3.2. Comparative Efficacy for Endoscopic Recurrence Prevention

The endoscopic recurrence prevention network incorporated 39 randomized trials (*n* = 3748 post-operative Crohn’s patients), with endoscopic recurrence predominantly defined as Rutgeerts’ score ≥ i2. Network geometry (Figure 2) demonstrated comparator selection patterns consistent with clinical relapse, predominantly utilizing placebo or thiopurine analogs as reference arms. Bayesian network meta-analysis (Figure 3, upper-right matrix) revealed significant efficacy hierarchy versus placebo: adalimumab monotherapy demonstrated superior protection (RR = 0.24, 0.11–0.47; SUCRA = 0.94; GRADE Low), followed by infliximab (RR = 0.32, 0.20–0.48; SUCRA = 0.86; GRADE Moderate), adalimumab/metronidazole combination (RR = 0.33, 0.13–0.79; SUCRA = 0.84; GRADE Very Low), azathioprine with exclusive enteral nutrition (RR = 0.35, 0.15–0.77; SUCRA = 0.81; GRADE Low), vedolizumab (RR = 0.36, 0.15–0.81; SUCRA = 0.80; GRADE Low), azathioprine/metronidazole combination (RR = 0.46, 0.26–0.78; SUCRA = 0.72; GRADE Very Low), and thiopurine analogs (RR = 0.64, 0.44–0.88; SUCRA = 0.71; GRADE Moderate), with complete SUCRA distributions shown in Figure 4b. Among the effective drugs, it was found that anti-TNF drugs had a significantly greater impact in preventing endoscopic recurrence compared to thiopurine analogs.

Node-splitting analysis identified significant local inconsistency only in the low-dose 5-ASA versus placebo comparison (*p* = 0.012, Supplementary Figure S1b), while all other contrasts showed acceptable coherence (*p* > 0.05). Substantial heterogeneity was observed (I^2^ = 62.8%), prompting meta-regression investigation. The male gender proportion exhibited negative modification effects on adalimumab plus metronidazole versus azathioprine plus metronidazole efficacy (β = 0.24, 0.08–0.77).

The results of the sensitivity analysis based on the frequentist approach are consistent with the above results obtained by the Bayesian method (Supplementary File S4).

The per-protocol (PP) analysis of 20 studies involving 1375 patients showed that the risk ratios (RRs) for drug efficacy were largely consistent with the intention-to-treat (ITT) analysis results. However, with the exception of infliximab (IFX), none of the other outcomes demonstrated statistically significant differences compared to placebo (Supplementary Figure S3).

Begg’s funnel plot analysis and Egger’s regression test (*p* = 0.06) did not reveal statistically significant asymmetry, suggesting no substantial evidence of publication bias across the included studies.

The evidence levels of all drug comparison results are summarized in Supplementary File S5.

### 3.3. Comparative Safety Profiles of Investigated Therapeutics

Network meta-analysis of treatment discontinuations attributed to adverse drug reactions demonstrated comparable tolerability profiles across therapeutic agents relative to placebo, with the notable exception of interleukin-10 (IL-10) demonstrating significantly elevated withdrawal risk (Supplementary Figure S4).

## 4. Discussion

### 4.1. Summary of Main Results

This study conducted a systematic review of existing randomized controlled trial (RCT) evidence. Through Bayesian network meta-analysis, adalimumab (ADA), infliximab (IFX), and thiopurine analogs were identified as demonstrating superior efficacy to placebo in preventing both clinical and endoscopic recurrence (*p* < 0.05 for all comparisons). Nitroimidazole derivatives and high-dose mesalamine exhibited partial effectiveness in clinical recurrence prevention, though with more modest effect sizes. Notably, short-term dual therapy regimens (ADA-nitroimidazole and azathioprine-nitroimidazole combinations) demonstrated enhanced clinical prevention compared to monotherapies. A single RCT investigation of vedolizumab revealed endoscopic benefit without corresponding clinical efficacy. Therapeutic rankings based on surface under the cumulative ranking curve (SUCRA) values are detailed in the Results section. Current evidence remains limited for newer biologics and small-molecule agents in postoperative Crohn’s disease management. In addition, we provided the results of the PP analysis in the Supplementary Materials. Safety analyses demonstrated no statistically significant increase in treatment discontinuation rates across all active interventions compared to placebo.

Robust evidence from three distinct meta-analytical approaches—RCT network meta-analysis [3], hybrid cohort–RCT network analysis [56], and anti-TNF-focused pooled studies [57]—has conclusively validated the clinical superiority of TNF-α antagonists (IFX and ADA). Network meta-analytical results indicated potential superiority of ADA over IFX in clinical recurrence prevention, though conflicting evidence emerged from direct comparative meta-analysis [58] that showed non-significant outcomes. Two critical methodological considerations explain these divergent conclusions: First, indirect comparison limitations in our study were evidenced by CINeMA assessment, revealing some concerns in “Within-study bias” and “Imprecision” domains (GRADE Low). Second, Gangwani, M.K. et al.’s incorporation of observational studies might have compromised internal validity through selection bias.

Our analytical approach uniquely prioritized evaluation of concomitant medication regimens, a dimension frequently neglected in prior research. The ADA-metronidazole combination demonstrated clinically meaningful superiority over both IFX monotherapy (GRADE Moderate) and nitroimidazole monotherapy (GRADE Moderate) in preventing clinical recurrence. This critical therapeutic interaction had not been systematically examined in previous network meta-analyses, which predominantly focused on monotherapy comparisons. Likewise, AZA-metronidazole combinations exhibited synergistic clinical benefits, achieving greater risk reduction than AZA monotherapy and improvement over metronidazole alone. This paralleled the ADA combination’s performance, suggesting a class effect advantage of nitroimidazole-based combination therapies in clinical recurrence prevention.

The therapeutic landscape for non-anti-TNF biologics remains understudied in postoperative recurrence management. Systematic review identified only one randomized controlled trial (RCT) evaluating vedolizumab [10], which demonstrated endoscopic efficacy comparable to anti-TNF agents, but ranked lower in the SUCRA hierarchy. The existing cohort study data regarding these therapeutic classes have been systematically synthesized in prior evidence reviews [56,59] and incorporated into current clinical guidelines [4]. Currently, no clinical investigations have been conducted to evaluate the efficacy of small-molecule agents in preventing postoperative recurrence of Crohn’s disease (CD). Only a solitary case report has been published documenting Janus kinase (JAK) inhibitor administration in a patient with post-surgical Crohn’s disease recurrence [60].

### 4.2. Overall Completeness and Applicability of Evidence

Our systematic review and network meta-analysis represent a comprehensive effort to evaluate the pharmacological strategies for preventing postoperative recurrence in Crohn’s disease. By systematically searching multiple databases up to 4 July 2024, we aimed to include all relevant randomized controlled trials (RCTs), regardless of language, publication date, or reporting format. This inclusive approach was designed to capture the full spectrum of available evidence, enhancing the generalizability of our findings to a diverse patient population with Crohn’s disease.

However, several limitations in the evidence base must be acknowledged. First, the included studies varied widely in terms of sample size, study design, and follow-up duration. Small sample sizes in some trials may have limited statistical power, leading to imprecise effect estimates and potentially missing true treatment effects. Second, the heterogeneity among studies was substantial in some analyses. For example, in the endoscopic recurrence prevention network, significant heterogeneity (I^2^ = 62.8%) was observed, which could be attributed to differences in patient characteristics, surgical procedures, and treatment regimens. Although we conducted meta-regression and subgroup analyses to explore the sources of heterogeneity, some of these factors may still confound the results.

In addition, the evidence for newer biologics and small-molecule agents in preventing postoperative recurrence of Crohn’s disease remains limited. Only one RCT was identified for vedolizumab [10], and no clinical investigations have been conducted for small-molecule agents. This lack of data restricts our ability to comprehensively evaluate the efficacy and safety of these emerging therapies.

The definitions of endoscopic recurrence in most studies are relatively consistent, with the Rutgeerts endoscopic score being greater than or equal to i2. However, when combined with the CINeMA analysis, issues such as within-study bias, imprecision, and heterogeneity still exist in the synthesized data. These problems result in some of the outcomes having a GRADE of low and very low. Regarding clinical recurrence, differences in definitions are a major concern. The definitions of outcome indicators for clinical recurrence vary significantly (for example, a Crohn’s Disease Activity Index (CDAI) score higher than 150–250 points, a 70-point increase compared to the baseline, etc., Supplementary Table S2). This may have caused detection biases and inconsistent reporting, thereby affecting the accuracy of our pooled estimates.

Despite these limitations, our meta-analysis provides valuable insights into the comparative efficacy and safety of different pharmacological interventions for preventing postoperative recurrence in Crohn’s disease. The results can assist clinicians in making more informed decisions when selecting appropriate preventive therapies for their patients. Future research should focus on conducting large-scale, well-designed RCTs to fill the existing evidence gaps, especially for newer therapies. Additionally, studies controlling for potential confounding factors and standardizing outcome measures are needed to generate higher-quality evidence and improve the precision of our understanding of the optimal pharmacological strategies for preventing postoperative recurrence in Crohn’s disease.

### 4.3. Implications of the Results for Practice, Policy, and Future Research

The clinical and policy implications of these findings necessitate multidimensional integration across practice guidelines, resource allocation, and research prioritization. In clinical management, anti-TNF agents (adalimumab/infliximab) and thiopurine analogs should form the therapeutic cornerstone for postoperative patients; their demonstrated efficacy in recurrence prevention outweighs potential risks when contextualized through individualized risk–benefit assessments—particularly regarding anti-TNF-associated infection susceptibilities and thiopurine-related hematologic toxicities. Concurrently, healthcare systems must operationalize these evidence hierarchies through dynamic reimbursement frameworks that balance biologic therapy costs against long-term disease burden reduction, while policy mechanisms should catalyze investigational investments addressing critical gaps in novel therapeutic validation. Research imperatives should prioritize multicenter confirmatory trials addressing knowledge gaps in novel biologics and small-molecule agents, while concurrently advancing longitudinal safety surveillance and mechanistic investigations.

Emerging evidence highlights artificial intelligence (AI) as a promising tool to refine postoperative recurrence prevention [61]. By integrating clinical phenotypes, endoscopic findings, and serological/microbiota data via machine learning, AI can accurately predict individual recurrence risk and assist in tailoring preventive therapies—complementing our established efficacy hierarchies. As emphasized in recent reviews, AI bridges population-level data (from our study) and personalized care, representing a critical direction for future translational research.

Collectively, these clinical, policy, and research strategies will enhance the implementation of our evidence-based therapeutic hierarchies, moving toward more precise, efficient, and patient-centered postoperative CD management.

## 5. Conclusions

This systematic review and network meta-analysis elucidate pharmacological strategies for preventing postoperative recurrence in Crohn’s disease. For clinical recurrence prevention, adalimumab demonstrates superior efficacy among monotherapies, followed by nitroimidazoles, infliximab, thiopurine analogs, and high-dose mesalamine. Combination therapies of ADA or AZA with nitroimidazoles show enhanced effectiveness compared to single-agent regimens. Regarding endoscopic recurrence prevention, the therapeutic hierarchy identifies adalimumab as most effective, followed by infliximab, vedolizumab, and thiopurine analogs. These evidence-based rankings inform clinical decision-making while highlighting critical knowledge gaps requiring confirmation through randomized controlled trials, particularly for newer biologics and small-molecule agents.

## Figures and Tables

**Table 1:** Basic information of included studies.

Author/ Publication Year	Country	Treatment Groups (I/C)	Sample Size, *n*	Age	Gender (Male, %)	Follow-Up Duration (Months)	Outcomes
Zhu W et al. 2015 [51]	China	Tripterygium glycosides 1.5 mg/kg/d AZA 2 mg/kg/d	45 45	33 32	66 71	12	CR + ER
Savarino E et al. 2013 [6]	Italy	ADA 160–80–40 mg q2W AZA 2 mg/kg/d 5-ASA 3 g/d	16 17 18	45 49 46	50 53 44	24	CR + ER
Rutgeerts P et al. 2005 [40]	Belgium	Ornidazole 1 g/d Placebo	38 40	35 30.5	42.1 50	12	CR + ER
Reinisch W et al. 2010 [49]	Europe	AZA 2.0–2.5 mg/kg/d 5-ASA 4 g/d	41 37	35.5 36	58.5 54.1	12	CR
Marteau P et al. 2006 [41]	France	L-johnsonii-LA1 2 × 109 CFU Placebo	48 50	32 29	54 42	6	CR + ER
Manosa M et al. 2012 [46]	Europe	Placebo 15–20 mg/kg/d + AZA 2–2.5 mg/kg/d Metronidazole 15–20 mg/kg/d + AZA 2–2.5 mg/kg/d	25 25	NA	NA	12	ER
López-Sanromán A et al. 2017 [7]	Spain	ADA 160–80–40 mg q2W + Metronidazole 250 mg tid AZA 2.5 mg/kg/d + Metronidazole 250 mg tid	45 39	35 37	42.2 59	12	ER
Liao N S et al. 2009 [44]	China	Tripterygium glycosides 60 mg/d SASP 4 g/d	21 18	36 34	57.1 50	12	CR + ER
Hirsch A et al. 2023 [9]	Israel	ADA 160–80–40 mg q2W 6-MP 50 mg/d- gradually increase the dose to 1.5 mg/kg/d	19 16	33.1 31.3	74 69	12	CR + ER
Hellers G et al. 1999 [38]	Sweden	Budesonide 6 mg/d Placebo	63 66	34 36	56 41	12	CR + ER
Fukushima K et al. 2018 [54]	Japan	IFX 5 mg/kg Induction + maintenance Placebo	19 19	36.6 37.6	89 68	24	CR + ER
Duan M et al. 2024 [55]	China	EEN for 3 months + AZA started within 2 weeks after surgery at 1 mg/kg/d in the first month and then increased to 2–2.5 mg/kg/d Normal diet + AZA started within 2 weeks after surgery at 1 mg/kg/d in the first month and then increased to 2–2.5 mg/kg/d	41 40	33 36	71 70	12	CR + ER
Desreumaux P et al. 2010 [45]	NA	Lactobacillus casei (Dn-114-Oo1) 6 × 1010 CFU/d Placebo	53 58	NA	NA	12	CR + ER
De Bruyn J R et al. 2018 [53]	Belgium and the Netherlands	Vitamin D 25,000 IU qw Placebo	72 71	34 37	38 40	6	CR + ER
D’Haens G R et al. 2008 [42]	Belgium	AZA (100 mg for weight < 60 kg, 150 mg for weight ≥ 60 kg) + Metronidazole 750 mg/d Metronidazole 750 mg/d	40 41	38.8 40	40 51	12	CR + ER
D’Haens G et al. 2023 [10]	Europe	VDZ 300 mg q8W Placebo	43 37	36 36	44 65	6	CR + ER
Cottone M et al. 2009 [43]	US	IFX 5 mg/kg Induction + maintenance Placebo	11 13	43 32	55 77	12	CR + ER
Caprilli R et al. 1994 [37]	Italy	5-ASA 2.4 g/d Placebo	47 48	35.5 33.7	68 48	12	CR + ER
Arnott I et al. 2016 [52]	UK	6-MP 1 mg/kg Placebo	128 112	39.2 38.2	38 40	36	CR + ER
Armuzzi A et al. 2013 [48]	Italy	AZA 2.5 mg/kg/d IFX 5 mg/kg Induction + maintenance	11 11	32 34	73 64	12	CR + ER
Ardizzone S et al. 2004 [39]	Italy	5-ASA 3 g/d AZA 2 mg/kg/d	71 71	NA	70 63	24	CR
Dumois R A et al. 2001 [20]	US	5-ASA 4 g/d Placebo	154 170	NA	NA	18	CR
De Bruyn J et al. 2019 [25]	NA	VitaminD 25,000 IU qw Placebo	72 71	34 37	38 40	6	CR + ER
Caprilli R et al. 2003 [21]	Italy	Mesalazine 4 g/d Mesalazine 2.4 g/d	101 105	33.8 36.4	49 61	12	CR + ER
Bergman L et al. 1976 [17]	Sweden	Corticosteroids (15 mg/d for 2 W–10 mg/d for 14 W–5 mg/d for 17 W) + SASP (3 g/d for 16 W–1.5 g/d for 17 W) Placebo	57 40	NA	51 51	32	CR
Lochs H et al. 2000 [28]	Europe	5-ASA 4 g/d Placebo	152 166	33.4 33.8	47 51	18	CR + ER
Hanauer S B et al. 2004 [31]	US	6-MP 50 mg/d 5-ASA 3 g/d	47 44	34.9 34.1	49 43	24	CR + ER
Ferrante M et al. 2014 [35]	US	AZA 2–2.5 mg/kg/d ED-AZA 2–2.5 mg/kg/d	32 31	NA	37.5 52	24	ER
Ewe K et al. 1989 [26]	German	SASP 3 g/d Placebo	111 121	32 30	43 54	36	CR + ER
Ewe K et al. 1999 [27]	German	Budesonide 3 mg/d Placebo	43 40	35 33	49 40	12	CR + ER
Colombel J F et al. 2001 [29]	Europe	Interleukin10 4 ug/kg/d Interleukin10 24 ug/kg/d Placebo	22 21 22	33 32 31	32 33 32	3	CR + ER
Bommelaer G et al. 2020 [36]	France	AZA 2.5 mg/kg/d + Curcumin 3 g/d AZA 2.5 mg/kg/d	31 31	35 37.6	19 48	6	CR + ER
McLeod, R. S. et al. 1995 [18]	Canada & USA	5-ASA 3 g/d Placebo	87 76	38.9 37.1	49.4 64.5	34.8 28.8	CR + ER
Mañosa, M. et al. 2013 [23]	Spain	AZA (2–2.5 mg/kg/d) + Metronidazole (15–20 mg/kg/d) AZA 2–2.5 mg/kg/d	25 25	36.2 34.5	48 60	12	ER
Herfarth, H. H. et al. 2013 [22]	USA	Ciprofloxacin 1 g/d Placebo	17 16	33 27	58.8 50	6	CR + ER
Florent, C. et al. 1996 [19]	France	5-ASA 3 g/d Placebo	65 61	35 32	35 54	3	ER
Fedorak, R. N. et al. 2015 [24]	Canada	VSL#3 2 sachets/d Placebo	58 62	37.6 35.9	51.7 51.6	3	ER
Tao, Q. S. et al. 2009 [33]	China	Tripterygium glycosides 60 mg/d 5-ASA 1 g/d	22 23	36 39	59.1 56.4	12	CR + ER
Ren, J. et al. 2013 [34]	China	Tripterygium glycosides 1 mg/kg/d 5-ASA 4 g/d	21 18	35 35	53.8 53.8	12	CR + ER
Regueiro, M. et al. 2016 [5]	USA	IFX 5 mg/kg q8W Placebo	147 150	35 34	52.4 54	18	CR + ER
Prantera, C. et al. 2002 [30]	Italy	Lactobacillus-GG 120 × 109 CFU Placebo	23 22	37.3 36.2	61 68	13	CR + ER
Yoshida, K. et al. 2012 [47]	Japan	IFX 5 mg/kg q8W Placebo	15 16	36.9 32.8	73.3 75	36	CR + ER
Tursi, A. et al. 2014 [50]	Italy	IFX 5 mg/kg Induction + maintenance ADA 160–80–40 mg q2W	10 10	34.5 30.5	40 50	12	CR + ER
Scapa, E. et al. 2018 [9]	Israel	ADA 6-MP	NA	NA	NA	NA	ER
Van Gossum, A. et al. 2007 [32]	NA	Lactobacillus johnsonii, 1010 CFU/d Placebo	34 36	38.7 35	56 50	3	ER

Abbreviations: AZA, Azathioprine; CR, Clinical Recurrence; ER, Endoscopic Recurrence; ADA, Adalimumab; 5 ASA, 5-Aminosalicylic Acid; CFU, Colony Forming Units; NA, Not Available; SASP, Sulfasalazine; 6-MP, 6-Mercaptopurine; IFX, Infliximab; EEN, Early Enteral Nutrition; VDZ, Vedolizumab; ED-AZA, Endoscopy Driven AZA. In the ED-AZA treatment group, patients received no pharmacological intervention during the initial 26-week observation period. The decision to initiate adjunctive azathioprine (AZA) therapy was determined by endoscopic recurrence assessment findings at week 26. Detailed treatment criteria and protocols should be referenced in the original study documentation. VSL#3—Each sachet contains 900 billion viable bacteria, comprising 4 strains of Lactobacillus, 3 strains of Bifidobacterium, and 1 strain of Streptococcus salivarius subspecies thermophilus.

## Data Availability

Please contact the corresponding author for data requests.

## Supplementary Materials

The following supporting information can be downloaded at: https://www.mdpi.com/article/10.3390/medicina62050883/s1. Supplementary Table S1. Search strategy. Supplementary Table S2. Clinical recurrence definition. Supplementary Figure S1. Node-splitting analysis assessing consistency between direct and indirect evidence in the network meta-analysis. (a) Node-splitting results for clinical recurrence prevention network. (b) Node-splitting results for endoscopic recurrence prevention network. *p*-values > 0.05 indicate no significant inconsistency between direct and indirect evidence. Supplementary Figure S2. Forest plots of fixed-effect model comparisons against placebo in the network meta-analysis evaluating postoperative pharmacological prophylaxis for clinical recurrence prevention in Crohn’s disease. Supplementary Figure S3. League table of comparative efficacy (per-protocol analysis) for postoperative recurrence prevention in Crohn’s disease. Drug names are positioned along the diagonal cells. Numerical values (risk ratios with 95% confidence intervals) in each cell indicate the comparative efficacy between the right-listed intervention and the upper-listed counterpart. The upper triangular matrix displays endoscopic recurrence outcomes, whereas the lower triangular matrix presents clinical recurrence outcomes. Blue-shaded cells denote statistically significant outcomes (*p* < 0.05). Supplementary Figure S4. League table of treatment discontinuation due to adverse drug effects in the network meta-analysis. Drug names are positioned along the diagonal cells. Numerical values (risk ratios with 95% confidence intervals) in each cell indicate the comparative efficacy between the right-listed intervention and the upper-listed counterpart. Blue-shaded cells denote statistically significant outcomes (*p* < 0.05). Supplementary File S1. ROB2. Supplementary File S2. CR-CINeMA-League. Supplementary File S3. CR-GRADE. Supplementary File S4. ER-CINeMA-Leagure. Supplementary File S5. ER-GRADE.

## Abbreviations

The following abbreviations are used in this manuscript:

CD	Crohn’s disease
RCTs	Randomized controlled trials
ITT	Intention-to-treat
PP	Per-protocol
RRs	Risk ratios
CIs	Confidence intervals
TNF-α	Tumor necrosis factor-alpha
POR	Postoperative recurrence
JAK	Janus kinase
PRISMA	Preferred Reporting Items for Systematic Reviews and Meta-Analyses
RoB2	Revised Cochrane Risk of Bias 2 tool
ADRs	Adverse drug reactions
GRADE	Grading of Recommendations Assessment, Development and Evaluation
SUCRA	Surface Under the Cumulative Ranking curve
MCMC	Markov chain Monte Carlo
VPCs	Variance partition coefficients
CINeMA	Confidence In Network Meta-Analysis
IQR	Interquartile range
IFX	Infliximab
ADA	Adalimumab
VDZ	Vedolizumab
UST	Ustekinumab
TG	Tripterygium glycosides
IL-10	Interleukin-10
CR	Clinical Recurrence
ER	Endoscopic Recurrence
AZA	Azathioprine
6-MP	6-Mercaptopurine
SASP	Sulfasalazine
EEN	Early Enteral Nutrition
ED-AZA	Endoscopy-driven AZA
5-ASA	5-Aminosalicylic Acid
CFU	Colony Forming Units
NA	Not Available
