# Supplementary material for: Pharmacological Strategies for Preventing Postoperative Recurrence in Crohn’s Disease: A Systematic Review and Network Meta-Analysis of Randomized Controlled Trials
Source: Medicina (Kaunas). 2026 May 5;62(5):883. doi: 10.3390/medicina62050883 (PMC13208836; doi:10.3390/medicina62050883)
Supplement: Supplementary file 1 [file medicina-62-00883-s001.zip › Supplementary Table S1 Search Strategy.docx]

Medline via Ovid

| # | Term |
| --- | --- |
| 1 | exp Crohn disease/ |
| 2 | Crohn*.mp. |
| 3 | IBD.mp. |
| 4 | Inflammatory bowel disease*.mp. |
| 5 | or/1-4 |
| 6 | exp Surgery/ |
| 7 | Surgery*.mp. |
| 8 | Surgery resection.mp. |
| 9 | Colectomy.mp. |
| 10 | Resection*.mp. |
| 11 | or/6-10 |
| 12 | Post operation.mp. |
| 13 | Post-operative.mp. |
| 14 | Post opera*.mp. |
| 15 | Postopera*.mp. |
| 16 | or/12-15 |
| 17 | random$.tw. |
| 18 | factorial$.tw. |
| 19 | (crossover$ or cross over$ or cross-over$).tw. |
| 20 | placebo$.tw |
| 21 | single blind.mp. |
| 22 | double blind.mp. |
| 23 | triple blind.mp. |
| 24 | (singl$ adj blind$).tw. |
| 25 | (double$ adj blind$).tw. |
| 26 | (tripl$ adj blind$).tw. |
| 27 | assign$.tw. |
| 28 | allocat$.tw. |
| 29 | randomized controlled trial/ |
| 30 | Or/17-29 |
| 31 | 5 and 16 and 30 |

Embase

| # | Term |
| --- | --- |
| 1 | random$.mp. |
| 2 | factorial$. mp. |
| 3 | (crossover$ or (cross NEXT/1 over$) or cross-over$). mp. |
| 4 | placebo$. mp. |
| 5 | single blind.mp. |
| 6 | double blind.mp. |
| 7 | triple blind.mp. |
| 8 | (singl$ NEXT/3 blind$). mp. |
| 9 | (double$ NEXT/3 blind$). mp. |
| 10 | (tripl$ NEXT/3 blind$). mp. |
| 11 | assign$. mp. |
| 12 | allocat$. mp. |
| 13 | ‘crossover procedure’/exp |
| 14 | ‘single blind procedure’/exp |
| 15 | ‘double blind procedure’/exp |
| 16 | ‘triple blind procedure’/exp |
| 17 | ‘randomized controlled trial’/exp |
| 18 | #1 OR #2 OR #3 OR #4 OR #5 OR #6 OR #7 OR #8 OR #9 OR #10 OR #11 OR #12 OR #13 OR #14 OR #15 OR #16 OR #17 |
| 19 | ‘crohn disease’/exp |
| 20 | crohn*.mp. |
| 21 | ibd.mp. |
| 22 | inflammatory bowel disease*.mp. |
| 23 | #19 OR #20 OR #21 OR #22 |
| 24 | ‘surgery’/exp |
| 25 | surgery*.mp. |
| 26 | surgery resection.mp. |
| 27 | colectomy.mp. |
| 28 | resection*.mp. |
| 29 | #24 OR #25 OR #26 OR #27 OR #28 |
| 30 | post operation.mp |
| 31 | ‘post-operative’.mp. |
| 32 | post opera*.mp. |
| 33 | postopera*.mp. |
| 34 | #30 OR #31 OR #32 OR #33 |
| 35 | #18 AND #23 AND #29 AND #34 |

Cochrane CENTRAL

| # | Term |
| --- | --- |
| 1 | MeSH: [Inflammatory bowel disease] explode all trees |
| 2 | Crohn Disease |
| 3 | Crohn |
| 4 | IBD |
| 5 | #1 or #2 or #3 or #4 |
| 6 | MeSH: [Colectomy] explode all trees |
| 7 | Surgery |
| 8 | Surgical* |
| 9 | Surgical resection |
| 10 | Resection* |
| 11 | #6 or #7 or #8 or #9 or #10 |
| 12 | Post operation |
| 13 | Post-operative |
| 14 | Post opera* |
| 15 | Postopera* |
| 16 | #12 or #13 or #14 or # 15 |

ClinicalTrials.gov/WHO ICTRP

| # | Term |
| --- | --- |
| 1 | Inflammatory bowel disease and surgery |
| 2 | Crohn’s disease and surgery |
| 3 | Inflammatory bowel disease and resection |
| 4 | Crohn’s disease and resection |
