# Supplementary material for: Pharmacological Strategies for Preventing Postoperative Recurrence in Crohn’s Disease: A Systematic Review and Network Meta-Analysis of Randomized Controlled Trials
Source: Medicina (Kaunas). 2026 May 5;62(5):883. doi: 10.3390/medicina62050883 (PMC13208836; doi:10.3390/medicina62050883)
Supplement: Supplementary file 1 [file medicina-62-00883-s001.zip › Supplementary Table S2 Clinical Recurrence Definition.docx]

Supplementary Table 2. Distribution of Clinical Recurrence Definitions in Included Trials

| Clinical Recurrence Definition | Number of Trials |
| --- | --- |
| CDAI＞150 | 11 |
| CDAI＞200 | 8 |
| CDAI＞220 | 3 |
| CDAI＞250 | 4 |
| CDAI increase ≥ 70 from baseline | 1 |
| HBI-based criteria (HBI ≥5, HBI ≥8, or HBI increase ≥3) | 3 |
| Other criteria (clinical symptoms requiring medical/surgical intervention, repeat surgery, fistula formation, septic complications and etc.) | 4 |
| Total | 34 |
