# Supplementary figures and images for: Pharmacological Strategies for Preventing Postoperative Recurrence in Crohn’s Disease: A Systematic Review and Network Meta-Analysis of Randomized Controlled Trials
Source: Medicina (Kaunas). 2026 May 5;62(5):883. doi: 10.3390/medicina62050883 (PMC13208836; doi:10.3390/medicina62050883)

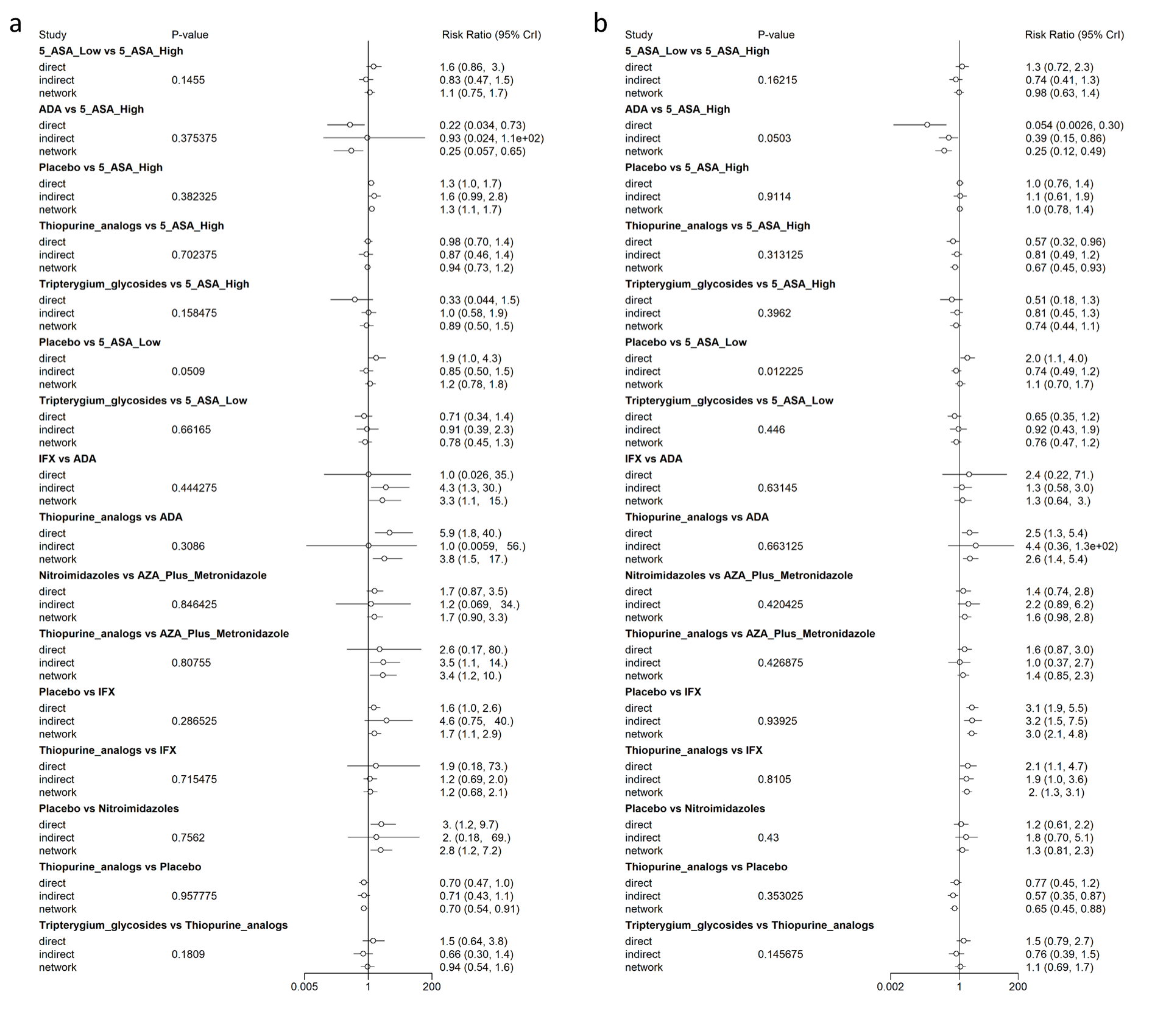

Supplement: Supplementary file 1 [file medicina-62-00883-s001.zip › Supplementary Figure S1 Consistency.tiff]

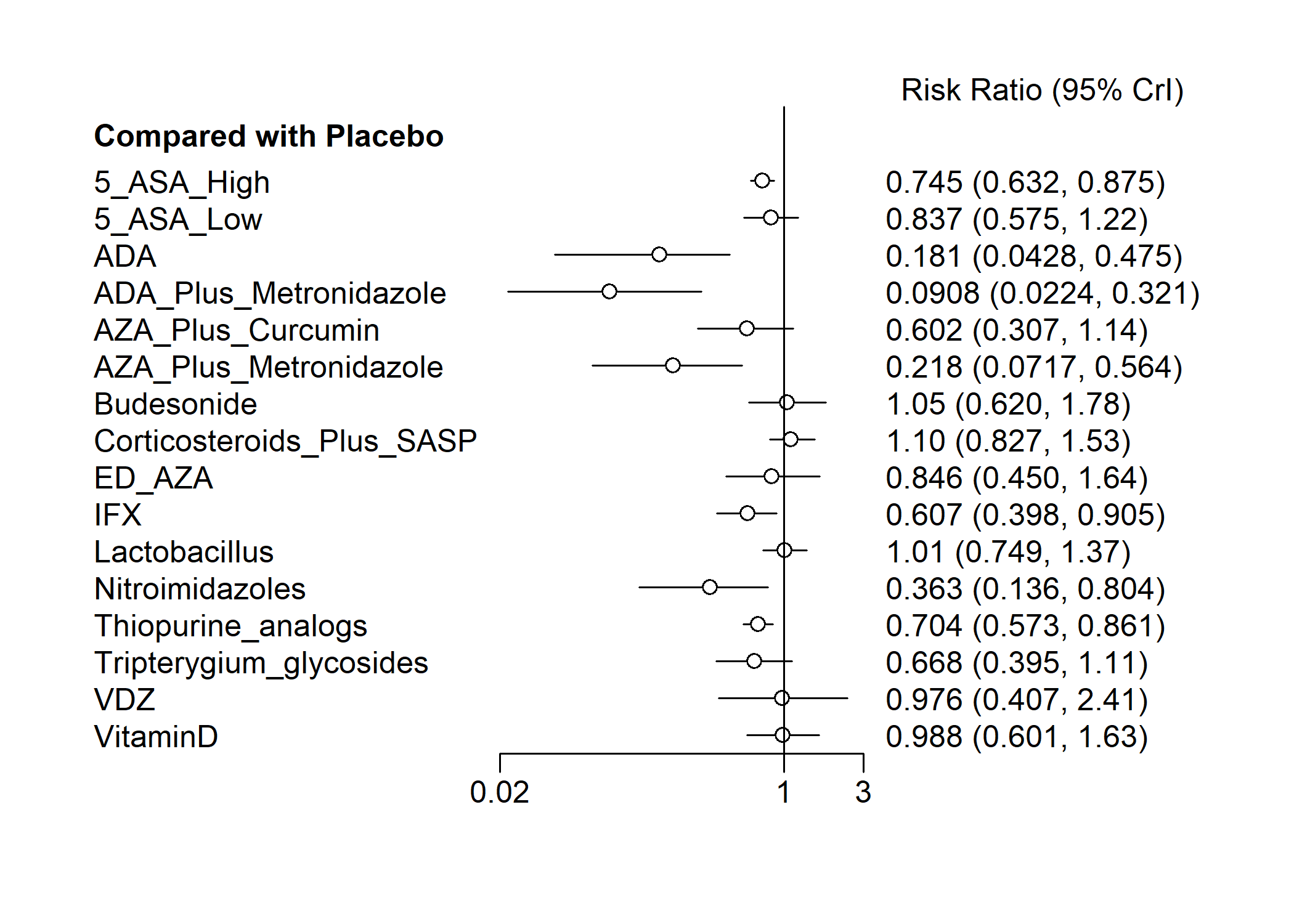

Supplement: Supplementary file 1 [file medicina-62-00883-s001.zip › Supplementary Figure S2 CRForest-fixed.tiff]
